# Alkali-Silica Reactivity of High Density Aggregates for Radiation Shielding Concrete

**DOI:** 10.3390/ma11112284

**Published:** 2018-11-15

**Authors:** Daria Jóźwiak-Niedźwiedzka, Michał A. Glinicki, Karolina Gibas, Tomasz Baran

**Affiliations:** 1 Institute of Fundamental Technological Research, Polish Academy of Sciences, Pawińskiego 5b, 02-106 Warsaw, Poland; mglinic@ippt.pan.pl (M.A.G.); kgibas@ippt.pan.pl (K.G.); 2 Institute of Ceramics and Building Materials, Cementowa 8, 31-983 Cracow, Poland; t.baran@icimb.pl

**Keywords:** alkali-silica reaction, heavyweight concrete, high-density aggregate, quartz size, radiation shielding, reactive minerals

## Abstract

Long-term exposure of concrete to nuclear reactor environments may enhance the ageing phenomena. An investigation concerning a possible deleterious alkali-silica reaction (ASR) in concrete containing high-density aggregates is presented in this paper. The scope of this investigation was limited to heavy aggregates that could be used for the construction of the first Polish nuclear power plant (NPP). Five different high-density aggregates were selected and tested: three barites, magnetite, and hematite. Mineralogical analysis was conducted using thin section microscopic observation in transmitted light. The accelerated mortar beam test and the long-time concrete prism test were applied to estimate the susceptibility of heavy aggregates to ASR. X-ray diffraction (XRD) and scanning electron microscopy (SEM) analyses were conducted on aggregates and mortars. The quartz size in aggregate grains was evaluated using image analysis. Application of the accelerated mortar beam method confirmed the observations of thin sections and XRD analysis of high-density aggregates. The microcrystalline quartz in hematite aggregate and cristobalite in one of barite aggregate triggered an ASR. The composition of ASR gel was confirmed by microscopic analysis. The long-term concrete test permitted the selection of innocuous high-density aggregates from among the other aggregates available, which showed practically no reactivity.

## 1. Introduction

Radiation shielding structures used in nuclear power plants are designed to withstand both mechanical loads and harsh environmental conditions, including elevated temperature exposure, thermal and humidity cycling related to fuel cycles, and long-term exposure to ionizing radiation. The normal service temperature range is up to 65 °C, and can be as high as 95 °C at localized hot zones [1,2]. The elevated temperature and high relative humidity (RH) in the concrete core of usually massive shielding structures may promote the development of a detrimental alkali-silica reaction (ASR), provided that both reactive minerals in the aggregate and sufficient alkali in concrete pore solution are present. The risk of ASR-induced damage in the concrete of nuclear power plants is not acceptable. Moreover, the long-term ionizing radiation from the reactor or other sources may further intensify the reaction in the nearby concrete elements [3]. Although the current knowledge of radiation-induced damage to concrete is still fragmented, it was suggested that quartz may exhibit increased chemical instability following neutron irradiation [4]. Quartz would eventually dissolve faster in the caustic concrete pore solution and therefore enhance the formation of alkali-silica gel provided that the other necessary conditions are met. ASR-related damage to concrete was detected in a nuclear power plant containment wall and an ageing management program was proposed [5]. The proper selection of concrete aggregates to be used in radiation shielding structures is of primary importance for this application.

Current aggregate selection procedures refer to ASR mitigation strategies defined in ASTM C1778 [6] and RILEM AAR-0 [7]. Nuclear power plant (NPP) safety-related structures are directly classified as “high risk” structures that require the highest level of ASR prevention. However, the materials selection is limited due to numerous requirements associated with radiation-shielding concrete [8,9]. For massive structures, the limitation of aggregate grain size is not applicable. The use of highly efficient supplementary cementitious materials in concrete, like siliceous fly ash, is questionable when considering potential activation of elements, which heavily influence the decommissioning time after the termination of the NPP service period.

Lee et al. [10] described petrographic examination methods that can be used to evaluate aggregates for use in radiation-shielding concrete. The authors stated that the aggregates used in this type of concrete should be relatively clean, free of deleterious materials, and chemically inert. The common types of potentially deleterious materials in aggregates include, among others, siliceous components of aggregates that are known to be associated with potentially harmful alkali-silica reactivity, so the aggregates should be examined to detect the presence of harmful minerals. Under the influence of ionizing radiation and elevated temperature in concrete shields, conditions for ASR promotion may occur [11]. Detailed mineralogical characterization and laboratory tests of special aggregates are thus necessary to prevent expansive alkali-silica reactions in concrete structures in NPP.

According to ACI 221R-96 [12], heavy fine, and coarse aggregates range in specific gravity from about 3500 to about 7500 kg/m^3^ and produce concrete ranging in unit weight from about 2800 to 5600 kg/m^3^. The aim of this work was the evaluation of high-density aggregates selected for radiation shielding concrete with respect to deleterious alkali-silica reactions. The scope of the investigation was limited to high-density aggregates that could be used for the construction of the first Polish NPP and that are economically reasonable. The cost of the aggregate, the expense involved in its transport, the aggregates properties, and availability of deposits were considered.

## 2. Materials and Methods

### 2.1. Materials

Naturally-occurring dense rocks includes high-density aggregates such as barite, magnetite, and other heavy metallic ores like hematite. Special high-density aggregates from the nearest available quarries were selected. Barite aggregates (B1, B2, and B3), a magnetite aggregate (M1) from European mines, and a hematite aggregate (H1) from Northern Africa were used. The density of the heavy aggregates was determined: 4.20 g/cm^3^ (B1), 4.05 g/cm^3^ (B2), 4.18 g/cm^3^ (B3), 4.80 g/cm^3^ (M1), and 5.10 g/cm^3^ (H1). Shielding concrete requires the applied high-density aggregate to display desirable protective properties against harmful radiation—primarily gamma radiation. In two analyzed barite aggregates, B1 and B3, the BaSO_4_ content was about 90%, but in barite aggregate B2, the content of BaSO_4_ was lower and was only ca. 78%. In the hematite and magnetite aggregate, the content of ferrum oxide was higher than 85%. The composition of the tested aggregates is presented in Table 1.

According to ACI Committee 221 [12], the exact chemical composition of high-density aggregates is not critical as long as the required density is met. However, pursuant to ACI Committee 304 [13], in some instances, the composition may be more important than the specific gravity of the high-density aggregate if the aggregate’s attenuation characteristics are good. According to the Polish Standard [14] concerning barite for special construction, the content of BaSO_4_ for special high-density concretes should amount to at least 75%. Ordinary Portland cement CEM I 42.5R was used in this research with alkali content Na_2_O = 0.12% and K_2_O = 0.78% (Na_2_O_eq_ = 0.63%) for the ASTM C1260 test [15].

Cement CEM I 42.5R with 0.9% Na_2_O_eq_ was used for the ASTM C1293 test [16]. Concrete mixes were created with a constant content of fine and coarse aggregate: 30% fine aggregate and 70% of coarse aggregate by volume. The content of cement paste was the same for each concrete mix. The same proportion of aggregate and cement mixtures was chosen to minimize their effect on the ASTM C1293 test results. The properties of concrete mixes and the compressive strength data are presented in Table 2.

Due to the possibility of the alkali release into pore solution as previously reported [17,18,19], the soluble alkali content was determined as shown in Table 3. A much higher water soluble alkali content was found in magnetite aggregate M1 than in hematite H1. However, the results of the ASTM C1260 test and microstructural analysis revealed the occurrence ASR in hematite aggregate. The content of the microcrystalline quartz had a stronger effect of the ASR (initiation and growing) than the soluble alkali content in aggregate.

### 2.2. XRD Analysis

The X-ray diffraction (XRD) method was used to analyse the mineral composition of the aggregates. All aggregates were powdered and sieved through a 0.045 mm sieve. A Bruker (Karlsruhe, Germany) D8 Discover diffractometer was used with a voltage ratio of 40 kV and 40 mA lamp current. A copper lamp was used as an X-ray source. The scan step size was 0.02°, collection time 1 s, and in the range 2Θ Cu Kα from 5 to 65° [21].

### 2.3. Thin Section

Thin sections impregnated with fluorescent epoxy were prepared according to Jóźwiak-Niedźwiedzka et al. [22]. The thin sections were prepared both from mortar bars and the aggregates. They were subjected to a process of impregnation with contrast-colored resin in a vacuum. The thin sections used for the microscopic analysis were 20 ± 2 μm thick. An Olympus (Olympus, Tokyo, Japan) BX51 petrographic microscope with a moving table and a digital camera was used. Photomicrographs were acquired using an Olympus DP25 digital color camera (Olympus, Tokyo, Japan), automatic moving table Prior ES11BX/B, (Prior Scientific Instruments Ltd., Cambridge, UK) and analySIS software (5.0, Olympus Soft Imaging Solutions GmbH, Munster, Germany). The transmitted polarized light with parallel nickel prisms (PPL), crossed nickol prism (XPL), crossed nickol prisms with gypsum plate (XPL_G), and ultraviolet (UV) light was applied.

### 2.4. ASTM C114

This standard covers specific chemical test methods that are grouped as reference test methods, among others, that refer to the determination of water-soluble alkalis. The test was performed on a small 25.0 g specimen, with 3 single specimens for each aggregate. The weighted specimen was put into a 500 mL Erlenmeyer flask and 250 mL of water was added. The obtained suspension was shaken continuously for 10 min at room temperature. Next, the specimen was filtered through a Buchner funnel into a 500 mL flask using a weak vacuum. Then, a 50 mL aliquot of the prepared solution was transferred to a 100 mL volumetric flask and the content of Na_2_O and K_2_O was determined using flame photometer.

### 2.5. ASTM C1260 Accelerated Mortar Bar Test

Accelerated Mortar Bar Test (AMBT) was performed on the standard specimens, but the duration of the test was prolonged up to 28 days according to the American Department of Defense (DOD) specifications [23]. These specifications rely primarily on ASTM C1260: each aggregate is first assessed against a threshold expansion of 0.08% at 28 days (of exposure). Three mortar bar specimens 25 × 25 × 285 mm were prepared for each aggregate, which were processed by crushing and sieving to the appropriate gradation. An aggregate-to-cement ratio of 2.15 and water-to-cement ratio (by weight) of 0.47 were maintained. After 24 h in the mold, the mortar bars were stored for the next 24 h in water in 80 ± 1 °C. After that, their initial zero readings were recorded by a digital extensometer before immersion in 1 M NaOH at 80 ± 1 °C. The subsequent measurements were recorded after 5, 7, 11, 14, 17, 21, and 28 days.

### 2.6. ASTM C1293 Concrete Prism Expansion Test

All the concrete prisms were prepared according to the above standard. A cement with an initial alkali content of 0.9% was used. The water-to-cement ratio was 0.45. The content of cement was 420 kg/m^3^ with the equivalent of Na_2_O adjusted to 1.25% of the cement mass, which resulted in 5.25 kg of alkali per 1 m^3^ of concrete. After casting prismatic 75 × 75 × 285 mm specimens, the specimens were protected from moisture loss and stored in the molds at 20 ± 2 °C for 1 day. Then, the prisms were demolded and stored in high humidity conditions at 38 ± 1 °C. The expansion result is an average of three specimens measured at 7, 28, 56, 90, 180, 270, and 365 days. An expansion limit of 0.04% at the end of the 1-year test was specified. The level of risk of alkali-silica reaction depends upon the importance of the concrete structure and the anticipated exposure conditions. In the radiation shielding concrete, ASR cannot be tolerated. Given the low risk tolerance for NPP structures, the class SC4 according to ASTM C1778 [6] was taken into consideration.

### 2.7. SEM-EDX Investigation

The specimens for scanning electron microscope-energy dispersive X-ray (SEM-EDX) investigation were obtained by slicing the mortar bars after completion of ASTM C1260 testing (28 days) with a slow speed diamond saw cooled by mineral oil. The specimens were then dried in an oven, vacuum-impregnated with a low-viscosity epoxy, lapped, and polished. Each specimen was prepared so that the polished face that was to be examined was a cut surface and two of the edges of this face of interest were originally exposed to the 1 M NaOH solution. Specimens were thoroughly examined using Nova NanoSEM 200 personal SEM (FEI Europe BV, Eindhoven, The Netherlands) in the backscatter mode using EDX microanalysis. An acceleration voltage of 20 kV was applied. 

### 2.8. Evaluation of Quartz Size in Aggregate Grains

To identify the content and size of quartz grains in heavy mineral aggregates, we analyzed the computerized image of the thin sections. Magnetite and hematite aggregates were included in the first step of the investigation because of their composition. In order to specify individual quartz crystals, the analysis was carried out with crossed nickol prisms and λ plate (XPL_G)—all the remaining minerals that contained iron oxide were opaque in the transmitted light. To analyze the size of individual quartz grains, the image analysis technique was used, in which the background was separated, i.e., opaque iron oxides and the resin used to prepare the thin sections, and then individual steps of the filtration operation were applied. The final classification of the quartz crystals was based on their average diameter size. According to the Norwegian Criteria, the classes of the quartz size were established [24]. Depending on the mean crystal size, the reactive forms of quartz were classified: highly-reactive quartz: <10 μm, reactive quartz: 10–60 μm, uncertain quartz: 60–130 μm, and harmless quartz: >130 μm. A description of the subsequent stages of the individual phases analysis is provided in Jóźwiak-Niedźwiedzka et al. [25].

## 3. Results

### 3.1. XRD and Petrographic Analysis

**Figure 1:**
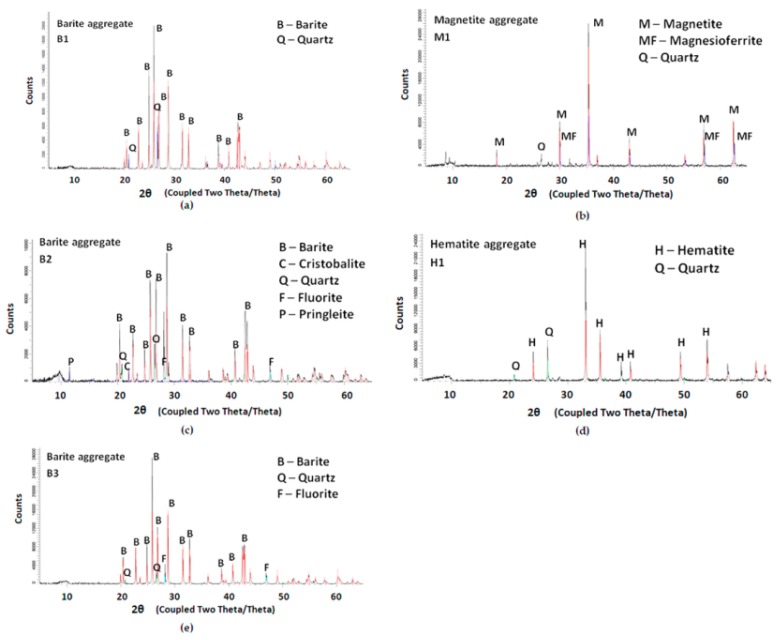
X-ray diffraction (XRD) patterns with main characteristic peaks in the tested high-density aggregates: (**a**) Barite B1, (**b**) Magnetite M1, (**c**) Barite B2, (**d**) Hematite H1, and (**e**) Barite B3.

XRD was used to characterize the main constitutive minerals in heavy aggregates (Figure 1). In all tested aggregates, the main peaks corresponded to the characteristic minerals for each type of aggregate: barite BaSO_4_, magnetite Fe_3_O_4_, and hematite Fe_2_O_3_. In each aggregate, the SiO_2_ peaks were visible as well as in aggregate B2—cristobalite, the natural form of metastable silica. Not all SiO_2_ crystals are prone to ASR and can cause reactions harmful to concrete [26].

**Figure 2:**
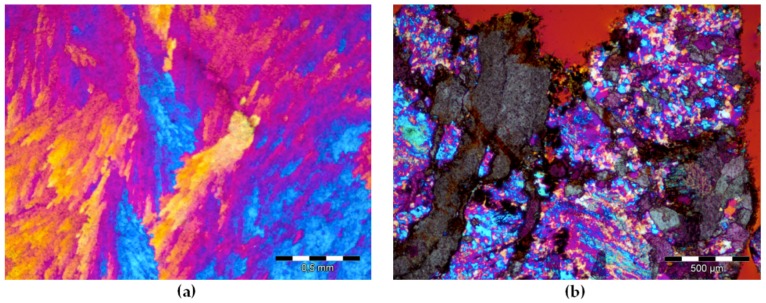
Barite aggregates in XPL_G: (**a**) B1 with characteristic plumose structure of BaSO_4_; (**b**) B2 with microcrystalline quartz and cristobalite; (scale bar = 500 µm).

**Figure 3:**
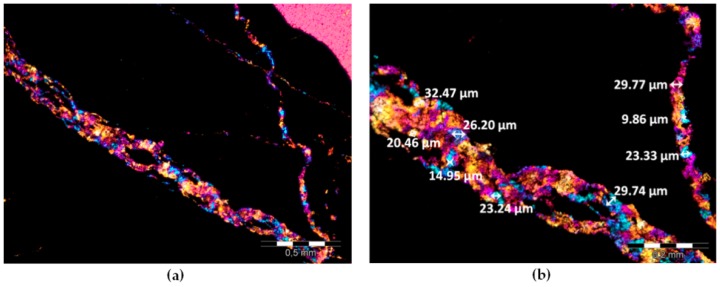
Hematite aggregate H1 with visible veins of microcrystalline quartz in XPL_G: (**a**) scale bar = 500 µm; (**b**) scale bar = 100 µm.

The petrographic analysis of the thin sections of the barite aggregates is described in detail in Jóźwiak-Niedźwiedzka et al. [27]. Barium sulphate crystals were the dominant mineral in aggregate B1, with few medium-grained SiO_2_ crystals. In barite aggregate B2, in addition to crystals of BaSO_4_ and SiO_2_, the cristobalite high-temperature polymorph of silica was also found. The presence of microcrystalline quartz was observed, with a crystal size equal to ca. 0.004–0.062 mm (Figure 2). Quartz can be considered microcrystalline if the crystal size is less than 100 μm and cryptocrystalline if less than 10 μm [28]. In the barite aggregate B3, not only barium sulphate was present, but also quartz (SiO_2_) and fluorite (CaF_2_). Magnetite and hematite aggregates contained mostly iron compounds, which were opaque in transmitted light. In magnetite aggregate, some SiO_2_ crystals were found but they were large enough to not promote ASR. In the hematite aggregate, small crystals of SiO_2_ were found (Figure 3). In order to properly assess the susceptibility of a given high-density aggregate to ASR, the content of reactive forms of SiO_2_ is crucial, not the total content of quartz. So, the thin section analysis was applied to estimate SiO_2_ crystals.

The hematite aggregate analyzed on thin section in XPL with gypsum plate is shown in Figure 3. The content of quartz crystals in the heavy aggregate was determined using the thin sections analysis technique. The content of quartz grains in the range of 10–60 μm in hematite aggregate was much higher than in the magnetite aggregate, which may suggest susceptibility to ASR. A previous investigation [25] demonstrated that the content of reactive SiO_2_ in hematite aggregate was 2.67% and only 0.13% in magnetite aggregate.

### 3.2. Expansion Tests and Post-Mortem Microstructure Evaluation

The properties of fresh mixes containing M1, B1, and B3 aggregate are presented in Table 2. The air content was low, about 1%. The bulk density of mixes differed depending on the aggregate used. The highest density was achieved for magnetite aggregate M1 due to the higher density of magnetite in relation to barite, 4.80 g/cm^3^ and 4.05–4.18 g/cm^3^, respectively. The compressive strength tested after 28 days of curing was within the range of 49 to 53 MPa. The highest value of f_c90_ was achieved for the concrete made with magnetite aggregate M1. Both the concrete compressive strength and the apparent density represent the properties of laboratory composition concrete as per ASTM C1293, which was loaded with extra alkalis. Using the same types of barite or magnetite aggregates, it was possible to produce heavyweight concrete with a bulk density of 3168–3317 kg/m^3^ [29].

The ASTM test methods, C1260 and C1293 [15,16], were applied to detect the potential for deleterious alkali-silica reaction in mortars and concrete prisms made with high-density aggregates. According to ASTM C1260, if after 14 days of testing the expansion of the mortar bars is lower than 0.10%, the aggregate may be considered non-reactive, and above 0.20% is highly reactive. According to the concrete prism test (ASTM C1293), the allowed expansion limit is 0.04%. The final results should be interpreted according to ASTM C1778 [6]. If an aggregate is potentially reactive based on the results of ASTM Cl260 (expansion >0.10% after 14 days immersion), its reactivity should be confirmed by testing using ASTM Cl293 or the potentially reactive aggregate should be used with appropriate mitigation measures. If the results of ASTM C1260 and ASTM C1293 are in disagreement, the results obtained using the ASTM Cl293 method should be considered more reliable.

**Figure 4:**
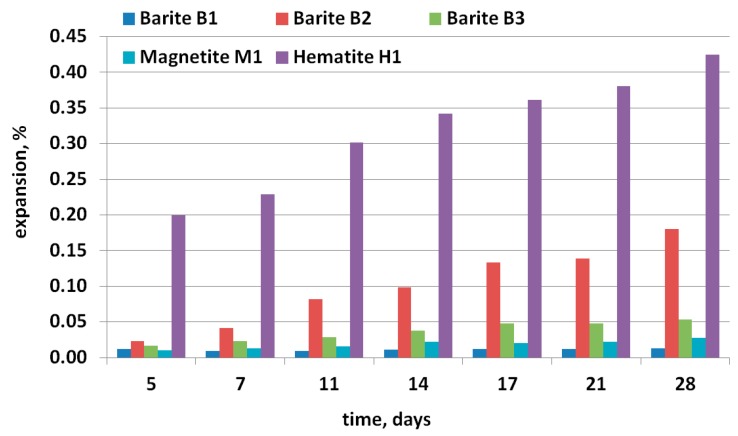
Expansion of mortar bars exposed to 1 M solution of sodium hydroxide and temperature of 80 °C.

Figure 4 presents the mortar bar test results according to ASTM C1260. Differences in the size of the beam expansion were related to the type of aggregate used. The expansion increased with the test duration. The fastest and the most extensive expansion development was found for hematite aggregate, and after four days, exceeded the 0.1% limit, reaching 0.43% after 28 days. The smallest bar expansion was achieved for barite aggregate B2 at 0.01%. After 14 days, all the bars made of hematite aggregate elongated well beyond the acceptable 0.1% (non-reactive aggregate) and between 0.10 and 0.30% (moderately reactive aggregate). They achieved an expansion of about 0.31–0.35%, which makes them highly reactive; class R2 is >0.30 and ≤0.45, according to AASHTO [30]. Other research indicated that a 0.08% limit would be more appropriate [31,32]. De Grosbois and Fontaine [33] suggested lowering the acceptable limits specification from 0.10% to 0.08%. According to the Department of Defense (DOD), the specification for the 28-day limit of mortar bar expansion is established at 0.08% [34]. Accepting all the above criteria, the three tested high-density aggregates, magnetite M1, and barites B1 and B3 met the requirements and did not show susceptibility to alkali-silica reaction. The risk tolerance should be extremely low for NPP applications and any mix design should be extremely conservative with regards to preventative measures (e.g., siliceous fly ash or very low alkali loading).

**Figure 5:**
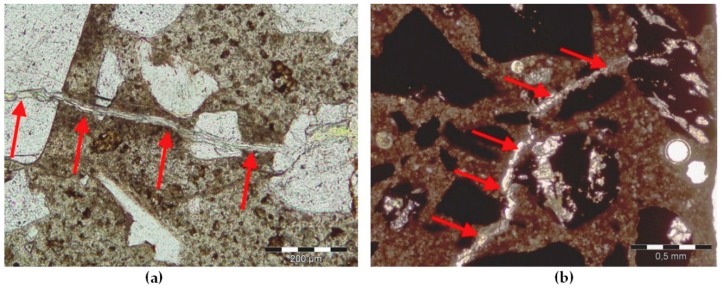
Post-mortem analysis on thin section in PPL, cracked mortar, and aggregate: (**a**) Barite B2, scale bar = 200 µm; (**b**) Hematite H1, scale bar = 500 µm.

**Figure 6:**
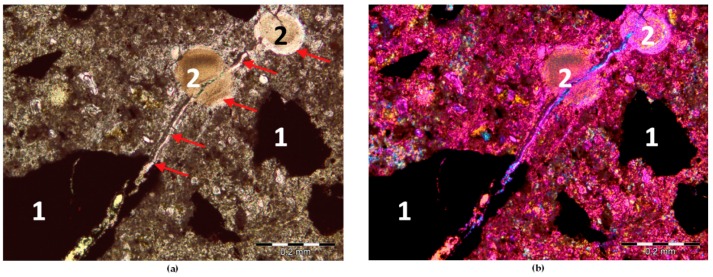
Post-mortem analysis on thin section, mortar with hematite H1: (**a**) PPL; (**b**) XPL_G; 1-hematite grains, 2-air-void filled by gel; scale bar = 200 µm.

**Figure 7:**
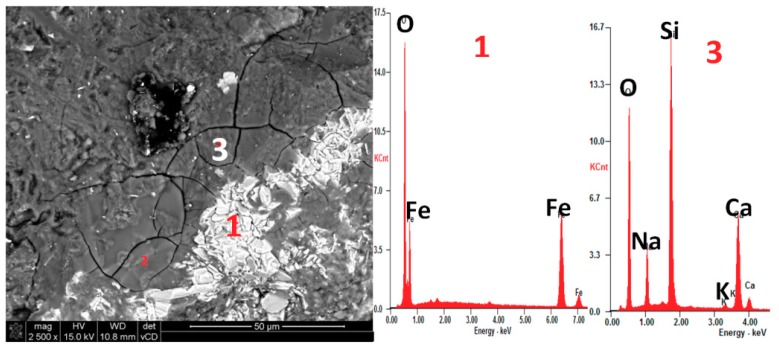
SEM microphotograph with EDX analysis showing the presence of (3) Si-Ca-Na gel in mortar bar with (1) hematite aggregate H1 after 28 days of exposure in 80 °C and 1 M NaOH.

**Figure 8:**
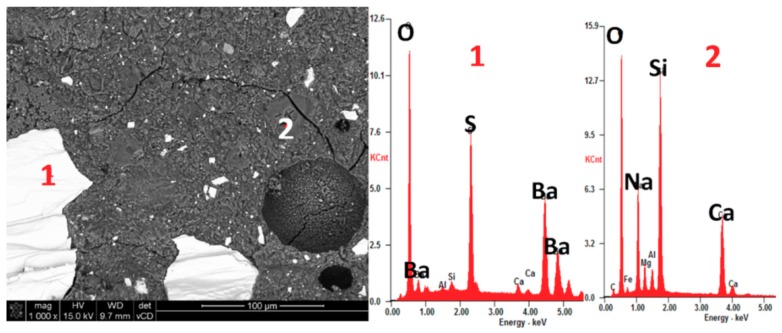
SEM microphotograph with EDX analysis showing the presence of (2) Si-Ca-Na gel in mortar bar with (1) barite aggregate B2 after 28 days of exposure in 80 °C and 1 M NaOH.

Identifying which constituent mineral of a rock is the cause of its reactivity is neither well understood nor evident for many types of rocks [28]. Therefore, we performed the post-mortem microstructure analysis using an optical microscope in transmitted light on thin sections (Figure 5 and Figure 6) and scanning electron microscopy (Figure 7 and Figure 8). The analysis on thin sections in transmitted light confirmed the presence of alkali-silica gel in the mortars made with barite B2 and hematite H1 aggregates. The cement matrix and aggregate were affected by gel. Figure 6 shows the gel that propagated from the hematite aggregate through the cement matrix and complete filling the air voids. The gel presence occurred as a result of the reaction of the quartz present in hematite aggregate; in the grains containing only hematite, no ASR occurred. The microcrystalline form of quartz, which was present in the barite and hematite aggregates, was deleterious to the occurrence of ASR. Figure 7 depicts the presence of the gel in both the aggregate and the cement matrix. In Figure 8, the Si-Ca-Na gel in mortar with barite aggregate B2 is clearly visible. In both cases, the sodium peaks are much stronger than the potassium peaks.

**Figure 9:**
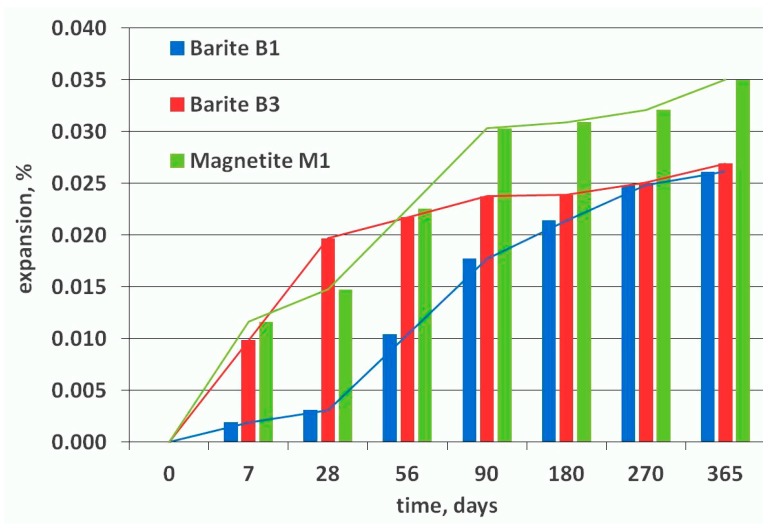
Expansion of concrete prisms stored in high humidity at temperature 38 °C over one year.

For the next step of the research, three high-density aggregates were chosen: magnetite M1 and barites B1 and B2, which positively passed the ASTM C1260 test. Figure 9 shows the expansion of the concrete prisms after one year of storage in 38 °C and high humidity in accordance with the ASTM C1293 test method.

During the first 56 days of testing, concrete prisms M1 and B3 expanded quickly, contrary to B1, which did not show large elongation at that time. The rate of increase in the length change for concretes M1 and B3 did not change significantly after 90 days. After one year, the concrete prisms made with barite aggregate, B1 and B3, showed similar results with expansion of ~0.026%, much below the allowable 0.04% limit. Concrete with magnetite aggregate M1 showed higher expansion than barite aggregates, at 0.035% after 365 days, but this is also below the allowable limit. The above results are partly opposite to the majority of the results published in the literature [35,36,37]. Most reactive aggregates show an initiation period with almost no expansion or even a slight shrinkage in the test. The typical expansion curve of a concrete prism affected by ASR can be characterized by three successive phases: an initial phase where swelling begins, followed by a phase of significant expansion at a nearly constant rate, and ending with a decrease in the expansion rate to reach a final plateau [36]. In this case, for concretes M1 and B3, the initial phase was not clearly separated from the expansion phase, and a final slope different from zero was finally observed.

Similar results were obtained in the AMBT test. Mortar bars with B3 barite after 14 days showed higher expansion than bars with B1 barite aggregate, and the increasing tendency was visible up to 28 days, whereas aggregate B1 seemed to reach a plateau.

## 4. Discussion

Petrographic analysis on thin sections revealed the presence of SiO_2_ crystals in all tested high-density aggregates. In hematite aggregate H1 and the barite aggregate B2, microcrystalline quartz was observed. In barite aggregate B2, cristobalite was found. Opposite results were reported by Topçu [38], who analyzed a barite aggregate from an unknown source using thin sections, but no mineral other than barite was found. Barite crystals showed dimensions that ranged between 25 and 1200 µm. Castro and Wigum [39] showed that image analysis petrography can be successfully used as a supplementary technique to overcome some of the limitations of the petrographic method RILEM AAR-1.

Several researchers [40,41] reported the results of mechanical tests on high-density concrete containing hematite aggregates from different sources. Gencel et al. [41] showed that mechanical properties, especially the compressive strength of concrete with hematite, did not differ from those of plain concrete. The influence of barite and magnetite aggregate on the permeability of radiation shielding concrete was reported by Kubissa et al. [29]. ACI limits the normal operating temperature in NPP concrete structures to 65 °C in general and 90 °C locally, so the exposure of concrete to high temperature is unavoidable, which may create favorable conditions for ASR. Pillai et al. [40] reported that, among all tested concretes, the concrete containing hematite aggregate showed better compressive strength than the reference concrete with granite aggregate after sustained thermal ageing for 56 days (84 MPa and 76 MPa, respectively). However, according to Jones and Clark [42], although the Young’s modulus of concrete can be significantly reduced by ASR, the apparent deterioration of concrete compressive strength due to ASR is dependent on the test method used. The cube test is particularly insensitive to ASR, with strengths after significant expansion often being greater than those at 28 days. So, the large expansion of the hematite H1 mortar and the threat of the ASR occurrence should be considered during the high-density concrete design process.

The mortar bar expansion test results showed that one barite aggregate was susceptible to ASR. The obtained results are in contrast to those of Pomaro et al. [43]. They stated that high specific weight aggregates like barites have almost null reactivity with alkalis in cement. It seems that the potential for ASR in barite aggregates is related to the content of secondary minerals, and particularly to the size and content of SiO_2_ (microcrystalline quartz). This is consistent with the review presented by Rajabipour et al. [44]. Aggregate reactivity depends not only on the type of silica mineral it contains, but also on the size and distribution of these minerals within the aggregate structure. However, Hagelia and Fernandes [17] suggested that the size of the quartz grains might be less important, and that the dissolution of feldspars and micas contribute to the reactivity. It was shown that products of the reaction were associated with coarse-grained quartz (up to 1500 μm in size and essentially free of deformation), whereas granitic mylonites containing microcrystalline quartz and low amounts of mica were found to be innocuous. However, according to Tiecher et al. [45], under favorable conditions of temperature, humidity, and access of alkali over a long time period, each type of quartz tends to provoke the alkali-silica reaction.

All the presented conclusions regarding reactivity or innocuousness are based only on laboratory tests. The data from real structures and from field exposure are unknown or cannot be published.

The results from the ASTM C1293 test confirm observations made by Islam et al. [46]. They showed that the mortar bars with hematite/magnetite submerged in 1 M NaOH expanded mostly during the early state of testing up to 28 days, and the expansion rate decreased with an increase in test duration. The concrete prism from barite (B1 and B3) and magnetite (M1) aggregates demonstrated the greatest elongation during the first days of testing up to 90 days. The sharp slope of the curve during the first 90 days may suggest a large potential for ASR, but after this time, the beams did not show such a rapid increase in length. Carles-Gibergues and Cyr [35] presented interpretation of expansion curves of concrete subjected to accelerated ASR tests. Besides the commonly known expansion curve of a concrete prism affected by ASR, which is characterized by three successive phases (an initial phase where swelling begins, a phase of significant expansion at a nearly constant rate, and a decrease in the expansion rate to reach a final plateau), the author showed another form of expansion curve. The initial phase was not clearly separated from the expansion phase. Nevertheless, all the curves clearly presented the same initial period—the highest expansion of prisms.

This research was limited to materials that could be used for the construction of the first Polish NPP and that are economically reasonable. There are no high-alkali cements available on the Polish market, so the tests were performed according to ASTM standards. According to ASTM C1260, the alkali content of the cement has a negligible or minor effect on the expansion in this test because the specimens are stored in a 1 M NaOH solution. Contrary to the RILEM guidelines for mortar bar, where the Na_2_O_eq_ content has to be 1%, the maximum level of Na_2_O_eq_ in Polish cement was equal to 0.88%, so met the ASTM C1293 requirements. In Jóźwiak-Niedźwiedzka et al. [27], the effect of alkali content in cement on the expansions due to ASR was studied. Two ordinary Portland cements (Na_2_O_eq_ = 0.8% and 0.6%), which are commonly available in the market, and one special cement were tested. One cement was specially made for the purpose of nuclear shielding concrete CEM I NA-SR-LH with low-alkali content, increased sulphate resistance, and low heat of hydration (Na_2_O_eq_ = 0.4%). For aggregates susceptible to ASR, the content of alkali in cement had no effect on the final test result, whereas for ASR-resistant aggregates, although the results of the expansion were lower than 0.1%, they differed depending on the amount of Na_2_O_eq_ in the cement. Finally, depending on the environmental conditions in which the concrete structure is expected to work, the choice of the appropriate cement should be considered.

## 5. Conclusions

We drew the following conclusions after our investigation into the alkali silica reactivity of high density aggregates:

(1) The thin section petrographic analysis and XRD analysis showed that the reactive silica in cristobalite form was present in barite aggregate (B2).
(2) The microcrystalline form of quartz, considered reactive, was identified in hematite aggregate (H1) and barite aggregate (B2).
(3) Reactive quartz grains sized 10 to 60 μm were detected in both high-density aggregates, but the content of the microcrystalline quartz was only 0.13% for magnetite aggregate (M1) and 2.67% for hematite aggregate (H1).
(4) According to the ASTM C1260 test, the highest expansion (0.34%) of mortar bars was found for hematite (H1) aggregate, and the smallest (0.03%) for specimens with magnetite (M1) aggregate.
(5) The presence of ASR gel in mortar bars with hematite (H1) and barite (B2) aggregate was confirmed in post-mortem analysis using SEM. The characteristic ASR composition of Si-Ca-Na gel was proven by the SEM-EDS analysis.
(6) Results obtained from the short-term (ASTM C1260) and the long-term (ASTM C1293) test methods permitted the selection of the high-density weight aggregates from among the options available, with no practical reactivity, required for applications in structures of major importance.
(7) The analysis of thin sections showed that aggregates that come from different mining sites, such as barite, vary significantly between in terms of mineral composition. It is important to consider not only the type of a rock as a criterion for its potential for reactivity, but also its mineralogical composition.

## Figures and Tables

**Table 1:** Main components of high-density aggregate after X-Ray Fluorescence (XRF) spectroscopy, in % by mass.

Aggregate	LOI	Main Mineral Constituent									
		SiO_2_	Al_2_O_3_	Fe_2_O_3_	CaO	MgO	SO_3_	Na_2_O	K_2_O	BaO	Mn_2_O_3_
Barite B1	1.10	4.88	0.15	0.26	2.39	0.14	30.23	0.04	0.00	59.26	0.06
Barite B2	11.54	9.26	1.06	21.31	0.17	1.28	17.68	0.05	0.23	35.48	1.15
Barite B3	2.05	3.56	0.73	5.52	1.58	1.05	27.59	0.04	0.01	57.23	0.04
Hematite H1	0.50	9.83	0.68	86.74	0.02	0.13	0.00	0.03	0.27	0.16	1.62
Magnetite M1	−2.42 ^1^	3.39	0.51	93.72	1.72	1.20	0.00	0.19	0.10	0.06	0.11

^1^ Weight increase associated with the formation of new oxides, LOI-loss on ignition.

**Table 2:** Properties of concrete mixes and the compressive strength after 28 and 90 days of curing.

Property	Magnetite M1	Barite B1	Barite B3
slump (mm)	50	130	50
temperature (°C)	22	22	22
bulk density (kg/m^3^)	2776	2696	2650
air content (%)	0.8	1.0	0.8
f_c28_ (MPa)	51.4	49.1	53.4
f_c90_ (MPa)	64.9	54.5	59.5

**Table 3:** Water soluble alkali content in high-density aggregates containing iron ore according to ASTM C114-13 [20] in mg/kg.

Soluble Alkali	Hematite H1	Magnetite M1
Na_2_O_soluble_	4.0	105.0
K_2_O_soluble_	1.5	162.0
Na_2_O_eqv-soluble_	5.0	211.0
